# Factors Influencing Delayed Treatment in Patients With Breast Cancer During COVID-19 Pandemic

**DOI:** 10.3389/fpubh.2022.808873

**Published:** 2022-04-29

**Authors:** Shengdong He, Yanlin Wang, Xiaoting Zhao, Fangying Xu, Juncheng Li, Tao Huang, Peng Sun, Lingfan Li, Xiang Ai, Hualin Xiao, Gang Xue, Siyi He

**Affiliations:** ^1^Department of Burn and Plastic Surgery, The General Hospital of Western Theater Command, Chengdu, China; ^2^Department of General Medicine, The General Hospital of Western Theater Command, Chengdu, China; ^3^Department of Cardiovascular Surgery, The General Hospital of Western Theater Command, Chengdu, China

**Keywords:** COVID-19, breast cancer, delayed treatment, anxiety, depression

## Abstract

**Background:**

The outbreak of coronavirus disease 2019 (COVID-19) has endangered human health and life. This pandemic has changed people's lifestyle and affected the regular delivery of standard cancer treatment. In the present study, we aimed to explore the influencing factors of delayed treatment in patients with breast cancer during COVID-19 pandemic.

**Methods:**

This study was a cross-sectional investigation, and the subjects were patients who were discharged from the department of burn and plastic surgery after February 2020. All participants completed this study's online questionnaire based on the WeChat and Wenjuanxing platforms. Levels of anxiety and depression were measured by the Hospital Anxiety and Depression Scale (HADS). Patients were divided into a delay group and non-delay group according to the occurrence of delayed treatment. Univariate analysis was performed by using the *t* test or chi-square test. A logistic regression model was employed to determine factors associated with delayed treatment.

**Results:**

The present study included a total of 397 patients with breast cancer, among whom delayed treatment occurred in 76 patients, accounting for 19.1%. Scores on both the anxiety subscale and depression subscale in delay group were significantly higher than those in non-delay group. Compared with non-delay group, we found that patients in delay group usually had a higher level of education (*P* = 0.020), worse self-feeling (*P* = 0.030), poor compliance of medical order (*P* = 0.042), and a higher prevalence of anxiety (*P* = 0.004) and depression (*P* = 0.012). Traffic inconvenience was also an important relevant factor for delayed treatment (*P* = 0.001). The prevalence of recurrence in delay group was higher than that in non-delay group (*P* = 0.018). By using logistic multivariate regression analysis, the results revealed that level of education and traffic inconvenience were independent factors influencing delayed treatment in patients with breast cancer during COVID-19 pandemic.

**Conclusion:**

The prevalence of delayed treatment in patients with breast cancer during COVID-19 pandemic is relatively high. Our findings reveal several influencing factors closely associated with delayed treatment, which is useful information that will be beneficial for patients to receive standardized therapy by taking targeted measures.

## Introduction

Since the outbreak of coronavirus disease 2019 (COVID-19), human health and life in hundreds of countries and regions around the world have been affected. This global public health event with widespread infection and difficult prevention has caused people to panic and experience anxiety. To control the spread of COVID-19, the Chinese government and medical institutions at all levels have taken effective measures to control the spread of COVID-19, such as reducing the number of people going out, enacting stay at home measures, encouraging appropriate personal protection, and strengthening measures regarding the quarantine of people and goods entering China, which have achieved remarkable results (1). However, with the discovery of multiple novel coronavirus variants, there are still new confirmed cases of COVID-19 in many regions, and the COVID-19 epidemic has entered a new pandemic phase (2).

According to an analysis of 2007 COVID-19 cases from 575 hospitals throughout China (3), cancer patients have a higher susceptibility to COVID-19 and are more likely to develop clinically severe events, with an incidence of more than three times that of the general population. Patients with breast cancer account for 17% of patients diagnosed with COVID-19 with a history of cancer (3). The susceptibility of breast cancer patients to COVID-19 is mainly due to low immunity. Recently, a few of studies have shown that people have been restricted from going out and exercising outdoors due to the impact of COVID-19, including cancer patients. These patients are more likely to suffer from delayed medical treatment and irregular treatment, as well as anxiety and other negative emotions, which could aggravate their illness (4–7).

Postoperative chemotherapy, radiotherapy, molecular targeted therapy and endocrine therapy for breast cancer patients all need to follow strict treatment norms. During the COVID-19 pandemic, delayed treatment for various reasons may lead to unsatisfactory efficacy, thus affecting patient's disease-free survival and overall survival (8, 9). For early-stage breast cancer, patients with delayed chemotherapy over 8 weeks after surgery had a higher risk of all-cause mortality than those with standardized chemotherapy (10). In China, people can buy government-provided medical insurance with very little money, including a new rural cooperative medical care system (NCMS) or basic medical insurance system for urban residents (BMIS-UR), which covers most of their medical expenses. However, there are still a small number of out-of-pocket expenses. At this time, people can buy commercial insurance as a supplement to reimburse what is not covered by government-provided medical insurance. The income of some breast cancer patients has decreased due to COVID-19, making it difficult for them to continue adjuvant cancer treatment (11). Therefore, the COVID-19 pandemic has had a significant impact on the physical and mental health of patients.

In the present study, we performed a cross-sectional investigation focusing on patients with breast cancer during the pandemic. We aimed to explore the prevalence of delayed treatment and identify the factors associated with delayed treatment, in order to provide better medical services for patients.

## Materials and Methods

### Study Setting

This study was a cross-sectional investigation, and the subjects were patients who were treated and discharged from the department of burn and plastic surgery after February 2020. The inclusion criteria were as follows: (1) patients with a definite diagnosis of breast cancer; (2) patients receiving treatment and follow-up visits during COVID-19 pandemic; (3) adult females older than 18 years; (4) patients who were discharged from the hospital. The exclusion criteria were as follows: (1) patients with severe cognitive disorders; (2) patients with multiple organ dysfunction syndrome. All patients gave informed consent and voluntarily participated in the survey.

### Data Collection

All participants completed the online questionnaire based on the WeChat and Wenjuanxing platforms (www.wjx.cn) from 4 August 2021 to 14 October 2021. The survey could be finished within 3 min. The basic information that was collected included age, stage of treatment, duration of disease, commercial insurance, types of medical insurance, monthly income, level of education, self-feeling, compliance of medical order, traffic inconvenience, hospital selection, recurrence and time for outdoor exercise. Levels of anxiety and depression were measured by the Hospital Anxiety and Depression Scale (HADS) developed by Zigmond and Snaith (12). At the time of discharging from hospital, we would inform patients in detail about the plan and time of treatment or follow-up in the following stage. In the questionnaire, we set the question as “Did delay occur at any stage of treatment or follow-up during this pandemic?” Delay or interruption in any treatment phase or follow-up would be considered as delayed treatment based on patient's self-report. When patients had any doubts in the process of filling in the questionnaire, our medical staff would give clear explanation to ensure the reliability of the results. Delayed treatment included the following types: (a) diagnosis was made but no surgery was performed; (b) surgery was performed but no adjuvant therapy was performed; (c) interruption of adjuvant therapy; (d) the adjuvant therapy was completed, but the follow-up examination was not conducted on time. Patients were divided into delay group and non-delay group according to the occurrence of delayed treatment. The scale consisted of 14 items, including 2 subscales of anxiety and depression with 7 items each. Each item was counted as 0–3 points, and the score range was 1–21 points. A score of 0 to 7 was determined to be asymptomatic, while a score of 8 to 21 could be judged as anxiety or depression.

### Statistical Analysis

Statistical analysis was performed using SPSS version 23.0 software. Normality of continuous variables was assessed by using the Kolmogorov-Smirnov test. Age was expressed as mean ± standard deviation (SD) because of a normal distribution, and *t* test was used for comparisons between delay group and non-delay group. The other measurement data were expressed as median (Q1–Q3), and the Mann-Whitney U test was used. The numerical data were expressed as *n* (%), and assessed with the chi-square test. The factors with differences in the univariate analysis were set as the covariates. With the occurrence of delayed treatment as the dependent variable, logistic multivariate regression analysis was performed. When the *P*-value was < 0.05, the difference was considered to be significant.

## Results

### General Information

A total of 458 patients were invited to participate, and the participation rate was 86.7%. The present study included a total of 397 patients with breast cancer, among which delayed treatment occurred in 76 patients, accounting for 19.1%. Patients in delay group had an average age of 50.5 ± 9.7 years, while patients in non-delay group were aged 48.5 ± 10.7 years. There were 52 (13.1%) patients who had had breast cancer <3 months, 130 (32.7%) patients who had had breast cancer 3–12 months, 182 (45.8%) patients who had had breast cancer 12–60 months, and 33 (8.3%) patients who had had breast cancer more than 60 months. The education level of the included patients was generally low, among which 203 cases (51.1%) had a middle school education or below and 72 cases (18.1%) had a bachelor's degree or above. Forty three cases (10.8%) had disease recurrence. Most of the patients stayed at home longer because of the epidemic, and 280 patients (70.5%) thought that the length of time for outdoor exercise was reduced.

### Mental Status of Patients With Breast Cancer During COVID-19 Pandemic

**Figure 1:**
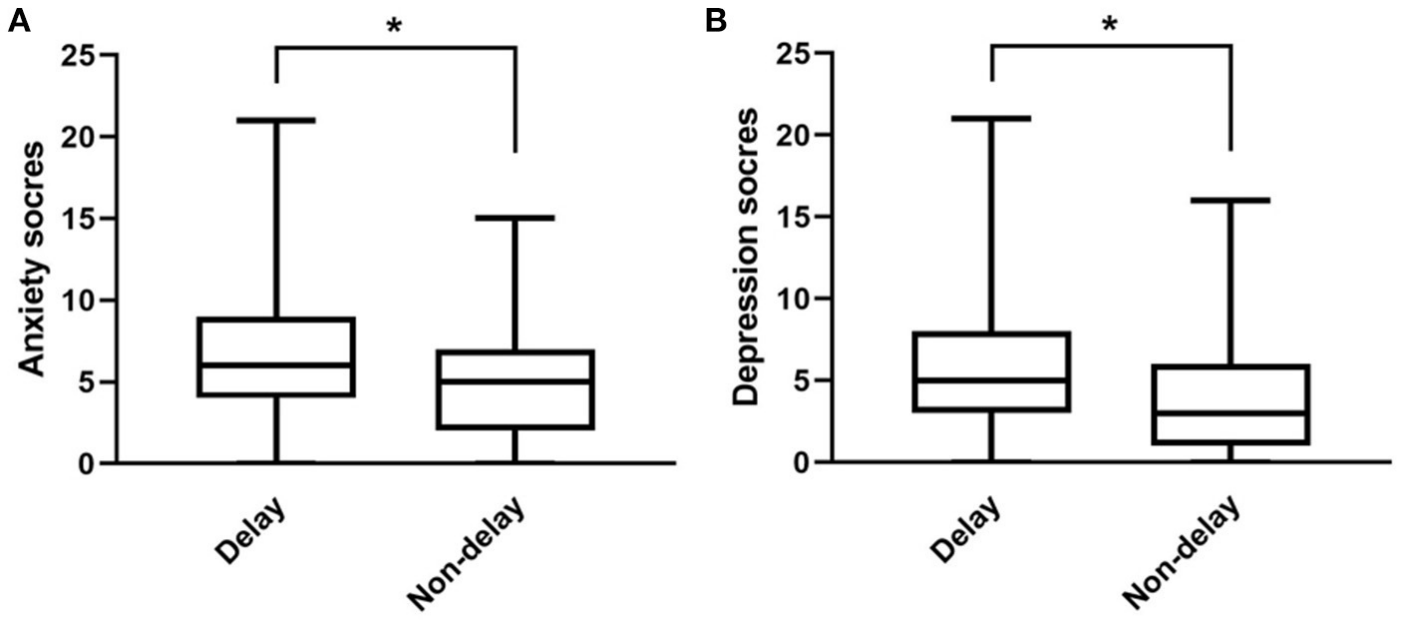
Comparison of anxiety scores **(A)** and depression scores **(B)** between delay group and non-delay group. Upper and lower transverse line represent the minimum and maximum value respectively. *P < 0.05.

Based on the evaluation criteria of the HADS, 64 cases (19.9%) were considered to have anxiety and 46 cases (26.3%) were considered to have depression in non-delay group. Once treatment was delayed, the prevalence rose to 35.5% (*P* = 0.004) and 26.3% (*P* = 0.012) respectively with a significant difference. Seventy six patients in delay group scored 6 (4–9) on the anxiety subscale and 5 (3–8) on the depression subscale, while 321 patients in non-delay group scored 5 (2–7) on the anxiety subscale and 3 (1–6) on the depression subscale. As shown in Figure 1, scores of both the anxiety subscale and depression subscale in delay group were significantly higher than those in non-delay group.

### Univariate Analysis of Influencing Factors for Delayed Treatment

Table 1 showed the results of univariate analysis. A detailed explanation of three subjective questions (self-feeling, compliance of medical order and traffic inconvenience) could be found in Supplementary Table 1. We found that patients in delay group usually had a higher level of education (*P* = 0.020), worse self-feeling (0.030) and poor compliance of medical order (0.042) compared with patients in non-delay group. Traffic inconvenience was also an important relevant factor for delayed treatment (*P* = 0.001). The prevalence of recurrence in delay group was higher than that in non-delay group (*P* = 0.018). Nevertheless, the items including age, stage of treatment, duration of disease, concerns about delayed treatment, commercial insurance, types of medical insurance and monthly income did not have significant differences between the two groups.

**Table 1:** Comparison of general information between two groups.

**Items**	**Delay group (*n* = 76)**	**Non-delay group** **(***n*** = 321)**	***P*** **-value**
Age (y)	50.5 ± 9.7	48.5 ± 10.7	0.125
Stage of treatment			0.796
Surgery	3 (3.9)	26 (8.1)	
Chemotherapy	12 (15.8)	51 (15.9)	
Radiotherapy	3 (3.9)	7 (2.2)	
Targeted therapy	18 (23.7)	78 (24.3)	
Endocrinotherapy	20 (26.3)	82 (25.5)	
Outpatient follow-up	20 (26.3)	77 (24.0)	
Duration of disease			0.223
<3 m	6 (7.9)	46 (14.3)	
3 m−1 y	22 (28.9)	108 (33.6)	
1–5 y	39 (51.3)	143 (44.5)	
>5 y	9 (11.8)	24 (7.5)	
Concerns about delayed treatment	16 (21.1)	71 (22.1)	0.839
Commercial insurance	7 (9.2)	43 (13.4)	0.323
Medical insurance			0.361
NCMS	18 (23.7)	83 (25.9)	
BMIS-UR	52 (68.4)	225 (70.1)	
self-paying	6 (7.9)	13 (4.0)	
Monthly income			0.566
<2,000 yuan	36 (47.4)	170 (53.0)	
2,000–5,000 yuan	25 (32.9)	97 (30.2)	
5,000–10,000 yuan	10 (13.2)	43 (13.4)	
>10,000 yuan	5 (6.6)	11 (3.4)	
Level of education			0.020*
Middle school or	36 (47.4)	167 (52.0)	
below			
Junior college	18 (23.7)	104 (32.4)	
Bachelor or above	22 (28.9)	50 (15.6)	
Self-feeling			0.030*
Well	49 (64.5)	235 (73.2)	
Uncomfortable	21 (27.6)	79 (24.6)	
Weak	6 (7.9)	7 (2.2)	
Compliance of medical order			0.042*
Good	70 (92.1)	311 (96.9)	
Fair	5 (6.6)	10 (3.1)	
Poor	1 (1.3)	0 (0)	
Traffic inconvenience	36 (47.4)	89 (27.2)	0.001*
Hospital selection			0.007*
Original treatment	48 (63.2)	224 (69.8)	
hospital			
Other tertiary	1 (1.3)	31 (9.7)	
hospital			
Nearest clinic	16 (21.1)	35 (10.9)	
Uncertainty	11 (14.5)	31 (9.7)	
Recurrence	14 (18.4)	29 (9.0)	0.018*
Anxiety	27 (35.5)	64 (19.9)	0.004*
Depression	20 (26.3)	46 (14.3)	0.012*

*NCMS, new rural cooperative medical care system; BMIS-UR, basic medical insurance system for urban residents*.

* *P < 0.05. Bold values mean the comparison is statistically significant*.

### Multivariate Analysis of Influencing Factors for Delayed Treatment

Nine factors with significant differences in the univariate analysis were further together analyzed by logistic multivariate regression analysis to acquire adjusted OR, including level of education, self-feeling, compliance of medical order, traffic inconvenience, hospital selection, recurrence, anxiety and depression (Table 2). When only the corresponding independent variable was entered into the model, we got the crude OR. For four polytomous variables (level of education, self-feeling, compliance of medical order, hospital selection), the first category was set as reference. Results revealed that level of education and traffic inconvenience were independent factors influencing delayed treatment in patients with breast cancer during COVID-19 pandemic. Compared with a middle school education or below, patients with a degree of bachelor or above were more susceptible to delayed treatment.

**Table 2:** Logistic regression analysis of independent influence factors for delayed treatment.

	**Crude OR (95% CI)**	**Adjusted OR (95% CI)**	***P*** **-value**
**Level of education**			
Middle school or below	Ref	-	**-**
Junior college	0.803 (0.433–1.487)	0.875 (0.451–1.697)	0.692
Bachelor or above	2.041 (1.101–3.784)	2.337 (1.192–4.581)*	**0.013**
**Self-feeling**			
Well	Ref	-	-
Uncomfortable	1.275 (0.720–2.257)	0.846 (0.433–1.652)	0.624
Weak	4.111 (1.324–12.764)	1.165 (0.302–4.499)	0.825
**Compliance of medical order**			
Good	Ref	-	-
Fair	2.221 (0.736–6.703)	1.595 (0.468–5.438)	0.455
Poor	7.177*10^9^ (0)	2.165*10^9^ (0)	1.000
Traffic Inconvenience	2.346 (1.406–3.916)	2.226 (1.262–3.927)*	**0.006**
**Hospital selection**			
Original treatment hospital	Ref	-	-
Other tertiary hospital	0.151 (0.020–1.130)	0.149 (0.019–1.163)	0.069
Nearest clinic	2.133 (1.093–4.163)	1.921 (0.918–4.018)	0.083
Uncertainty	1.656 (0.778–3.524)	1.661 (0.753–3.663)	0.208
Recurrence	0.440 (0.220–0.881)	0.610 (0.269–1.385)	0.237
Anxiety	2.213 (1.285–3.811)	1.548 (0.751–3.192)	0.237
Depression	2.135 (1.174–3.884)	1.437 (0.653–3.161)	0.368

*OR, odds ratio; CI, confidential interval*.

* *P < 0.05. Bold values mean the comparison is statistically significant*.

## Discussion

The present cross-sectional study enrolled 397 patients with breast cancer discharged from the department of burn and plastic surgery during COVID-19 pandemic, of which 76 patients reported delayed treatment. Delayed treatment was found to be closely associated with a high proportion and scores of both anxiety and depression. Based on univariate and logistic multivariate regression analysis, level of education and traffic inconvenience were independent factors influencing delayed treatment.

Many of the patients with severe COVID-19 are elderly individuals or having underlying conditions. The immune function of patients with tumors is decreased due to tumor growth, malnutrition and antitumor therapy, leading to increased susceptibility to COVID-19 and a higher proportion of critically ill patients. With the outbreak of public health hazards caused by COVID-19, the government and medical institutions at all levels took multiple measures to control the epidemic, such as limiting the flow of personnel, closing some businesses, halting production in factories, and strengthening personal health education. All these precautions have been taken to effectively curb the further spread of the epidemic. The present study found that the outdoor exercise time of patients during COVID-19 pandemic was significantly reduced, which was closely related to patient's high safety awareness and strict management of prevention and control. This was also the main reason for the delay in medical treatment. A meta-analysis pointed out that for every 4 weeks of delayed chemotherapy in patients with breast cancer, the risk of death would increase by 15% (13). In addition, Bleicher et al. (14) found that the survival time of patients decreases due to the delay of operations. Therefore, it is of great clinical significance to address delayed treatment during the pandemic.

Among the 397 cases of breast cancer in our investigation, 76 patients had delayed medical treatment, with a prevalence of up to 19.1%. According to the results of investigation, 78.1% of the patients expressed concerns about the impact of delayed treatment on the condition of their illness to various degrees. However, 78.8% of the patients were more willing to receive antitumor treatment in the original hospital. This result suggested that patients did not have a negative attitude regarding therapy, although they were affected by the epidemic and still hoping for better medical care. Multivariate analysis showed that a high level of education and traffic inconvenience increased the prevalence of delayed treatment. Patients with higher education levels may be more conscious of safety and overinterpret the dangers of COVID-19. Various control measures during the epidemic, such as home quarantine and road closures, makes it more difficult for patients who experience traffic inconvenience to access hospitals for treatment, resulting in delayed treatment.

Studies have shown that many patients are worried about delayed treatment. The delay of treatment can lead to an increase in depression and anxiety in patients with cancers (15, 16), which is consistent with our findings. Psychological factors could not be neglected in the progression of breast cancer. Anxiety, depression and other emotions also influence the diagnosis and treatment of patients with breast cancer (17, 18). A recent study found that the prevalence of depression among breast cancer patients was 9%, which could be affected by factors such as stages of disease and treatment (19). The occurrence of anxiety and depression would indeed increase during the period of disease therapy (20). In our research, the percentages of anxiety and depression in breast cancer patients were 35.5 and 26.3% respectively during the COVID-19 pandemic, which were significantly higher than those in previous literature before the epidemic. These higher percentages may be due to concerns regarding COVID-19 infection. On the other hand, they may be related to the fact that most patients worry that delayed treatment will affect their health conditions. Anxiety about the epidemic will discourage patients from going out to seek medical care. Depression could make patients afraid to face the disease. A passive and evasive attitude will lead to the loss of initiative for treatment. Patients with psychological problems need to be treated by specialists in a timely manner, and early identification and intervention could improve the prognosis of patients (21). In addition, light symptoms of depression and anxiety are closely associated with the late stage of treatment. Burgess and his colleagues demonstrated that the prevalence of anxiety and depression in patients with breast cancer was 50% at 1 year, 25% at 2–4 years, and 15% at 5 years after diagnosis, suggesting that anxiety and depression gradually disappeared over time (22). Physical health problems and psychological health problems often occur together. If psychological problems are not treated in time, the psychological state of anxiety and depression may promote the further development and deterioration of physical diseases (23, 24).

The limitation of this study is that it is only a cross-sectional investigation in a single center. There is no vertical comparison between the factors related to the delay before and after the outbreak of COVID-19, so the study cannot accurately evaluate the severity of delayed treatment. The judgement of delayed treatment is mainly based on patient's self-report, so individuals may perceive delay differently. Another limitation is that TNM stage information was not collected in our study. Patients at different stages of cancer will receive different treatments, and the delay or interruption of treatment could make a difference. Therefore, more studies with complete information are needed to clarify the impact of public health emergencies on the delayed treatment of patients with breast cancer.

Patients with tumor mostly experience fear and anxiety due to their own disease. Physical dysfunction or discomfort caused by the disease itself will further aggravate the psychological disorders of patients. For patients with delayed treatment, it is important to strengthen the standardized treatment of breast cancer and education on COVID-19. Furthermore, timely and necessary psychological intervention is also needed to relieve anxiety and depression, thus improving mental status. New methods of consultation, such as online consultations *via* cellphones, are worth popularizing during the COVID-19 pandemic. This will reduce the movement of people and ensure timely medical treatment at the same time for prevention and control of the epidemic.

## Conclusion

The prevalence of delayed treatment in patients with breast cancer during COVID-19 pandemic is relatively high, which is closely related to adverse psychological reactions of anxiety and depression. Taking targeted measures based on influencing factors will be beneficial for patients to receive standardized therapy. Timely and effective psychological intervention, as well as rational allocation of medical resources, will provide patients with better medical services and improve clinical prognosis. Our findings could motivate medical institutions to offer psychosocial support for patients, and serve as an important guide to develop treatment protocols for breast cancer during COVID-19 pandemic.

## Data Availability Statement

The raw data supporting the conclusions of this article will be made available by the authors, without undue reservation.

## Ethics Statement

Ethical review and approval was not required for the study on human participants in accordance with the local legislation and institutional requirements. The patients/participants provided their written informed consent to participate in this study.

## Author Contributions

SDH, GX, and SIH conceptualized and designed the study. YW, XZ, PS, LL, and FX undertook data collection. SDH, JL, and TH performed statistical analysis. SDH, XA, and HX wrote the manuscript. GX and SIH revised the manuscript. All authors contributed to the article and approved the submitted version.

## Conflict of Interest

The authors declare that the research was conducted in the absence of any commercial or financial relationships that could be construed as a potential conflict of interest.

## Publisher's Note

All claims expressed in this article are solely those of the authors and do not necessarily represent those of their affiliated organizations, or those of the publisher, the editors and the reviewers. Any product that may be evaluated in this article, or claim that may be made by its manufacturer, is not guaranteed or endorsed by the publisher.
