# Supplementary material for: Factors Influencing Delayed Treatment in Patients With Breast Cancer During COVID-19 Pandemic
Source: Front Public Health. 2022 Apr 29;10:808873. doi: 10.3389/fpubh.2022.808873 (PMC9099139; doi:10.3389/fpubh.2022.808873)
Supplement: Supplementary file 1 [file Table_1.DOCX]

**Supplementary Table 1.** Explanation of three subjective questions.

| Self-feeling |  |
| --- | --- |
| Well | Patients feel normal and have no discomfort. |
| Uncomfortable | Patients feel uncomfortable occasionally. |
| Weak | Patients always feel uncomfortable. |
| Compliance of medical order |  |
| Good | Patients strictly follow the doctor's advice for treatment and follow-up. |
| Fair | Patients partly follow the doctor's advice for treatment and follow-up. |
| Poor | Patients do not follow the doctor's advice for treatment and follow-up. |
| Traffic |  |
| Convenience | The distance is short, and there are optional means of transportation which can directly reach the medical institution. |
| Inconvenience | The distance is long, and there are no means of transportation which can directly reach the medical institution. |
